# Association of epicardial adipose tissue with serum level of cystatin C in type 2 diabetes

**DOI:** 10.1371/journal.pone.0184723

**Published:** 2017-09-18

**Authors:** Tomomi Murai, Noriko Takebe, Kan Nagasawa, Yusuke Todate, Riyuki Nakagawa, Rieko Nakano, Mari Hangai, Yutaka Hasegawa, Yoshihiko Takahashi, Kunihiro Yoshioka, Yasushi Ishigaki

**Affiliations:** 1 Division of Diabetes and Metabolism, Department of Internal Medicine, Iwate Medical University, Morioka, Japan; 2 Division of Cardiovascular Radiology, Department of Radiology, Iwate Medical University, Morioka, Japan; The University of Hong Kong, HONG KONG

## Abstract

**Objective:**

Accumulation of epicardial adipose tissue (EAT) is considered to be a cardiovascular risk factor independent from visceral adiposity, obesity, hypertension and diabetes. We explored the parameters related to EAT accumulation, aiming to clarify the novel pathophysiological roles of EAT in subjects with type 2 diabetes (T2DM).

**Methods:**

We examined the laboratory values, including cystatinC, and surrogate markers used for evaluating atherosclerosis. EAT was measured as the sum of the adipose tissue area, obtained by plain computed tomography scans in 208 subjects with T2DM but no history of coronary artery disease.

**Results:**

EAT correlated positively with age, body mass index (BMI), visceral fat area, leptin, cystatin C and C-peptide, while correlating negatively with adiponectin, estimated glomerular filteration rate (eGFR) and the liver-to-spleen ratio. Multiple linear regression analysis revealed serum cystatin C (β = 0.175), leptin (β = 0.536), BMI (β = 0.393) and age (β = 0.269) to be the only parameters showing independent statistically significant associations with EAT. When cystatin C was replaced with eGFR, eGFR showed no significant correlation with EAT. In reverse analysis, serum cystatin C was significantly associated with EAT after adjustment in multivariate analysis.

**Discussion:**

EAT accumulation and elevated cystatin C have been independently regarded as risk factors influencing atherosclerosis. The strong association between EAT and cystatin C demonstrated herein indicates that EAT accumulation may play an important role in Cystatin C secretion, possibly contributing to cardiometabolic risk in T2DM patients.

## Introduction

Epicardial adipose tissue (EAT) has recently been recognized not only as fat deposited around the pericardium, but also as a metabolically active tissue, secreting various humoral factors [1]. The volume of EAT accumulation is, in fact, associated with parameters related to obesity, especially the visceral fat area, as well as markers of insulin resistance [2]. In this decade, the pathophysiological roles of EAT have been attracting attention based on their relevance to both atherosclerotic surrogate markers [3, 4] and cardiovascular disease [5, 6], independent of obesity, hypertension and type 2 diabetes mellitus (T2DM). Moreover, a case-control study showed increased EAT volume to be related to major adverse cardiac events in subjects who have no prior history of coronary artery disease (CAD) [7].

EAT is a source of bioactive molecules, including adipocyokines and growth factors, directly impacting inflammation of the myocardium and coronary arteries. Similar to visceral fat, increasing obesity accompanied by epicardial adipocyte enlargement, leads to deterioration of adipocytokine signaling, including enhancement of plasminogen activator inhibitor (PAI)-1, tumor necrosis factor (TNF)-α and leptin expressions and a decrease in adiponectin expression [8]. This potential for local production of various cytokines is regarded as a major mechanism underlying the effects of EAT accumulation on the development of atherosclerosis. The profile of adipocytokine expression in EAT is reportedly comparable to that in visceral fat, as exemplified by the reduced expression of adiponectin in adipose tissue from CAD subjects [9].

The recent advances in imaging technology enable us to quantify EAT using modalities such as echocardiography [10], magnetic resonance imaging [11] and multi-detector computed tomography (MDCT) [12]. Among these modalities, MDCT provides the most reproducible determination of EAT because of its higher spatial resolution, resulting in accurate quantification. Obtaining information on calcification of coronary arteries, an established predictor of CAD, is another merit of performing cardiac CT.

While several studies have shown the importance of EAT accumulation in the development of atherosclerosis, the EAT associated factors influencing atherosclerosis as not as yet fully understand. Thus, we designed this cross-sectional study to examine associations among EAT, humoral factors and atherosclerotic surrogate markers in T2DM patients, who are known to be at risk for CAD. In this study, we explored the parameters related to EAT accumulation, aiming to clarify the novel pathophysiological roles of EAT in subjects with T2DM.

## Materials and methods

### Study subjects

The study subjects were T2DM patients admitted to Iwate Medical University Hospital during the period from January 2014 to July 2016. Two hundred and eight subjects, all of whom underwent cardiac MDCT, were enrolled in this study. Patients were excluded if they had renal dysfunction (estimated glomerular filtration rate with serum creatinine [eGFRcre] below 45 mL min^-1^ 1.73 m^-2^), any malignancy, an infectious disorder, collagen disease or a past history of CAD. This study was approved by the Institutional Review Board of Iwate Medical University (Approval number: H27-30). The concent of the study was informed and obtained by written form.

### Quantification of EAT

EAT and the coronary artery calcification score (CACS) were quantified on ECG-gated diagnostic cardiac CT scans with some modification [13, 14]. A VCT 240 slice MDCT (Aquillion ONE, Toshiba Medical, Tokyo, Japan) was used to obtain plain multi-slice CT scans, performed with a 0.5 mm collimation width, a gantry rotation speed of 0.4 s/rotation, 120 kV and 300 mA, using prospective ECG-gated axial scanning.

**Fig 1:**
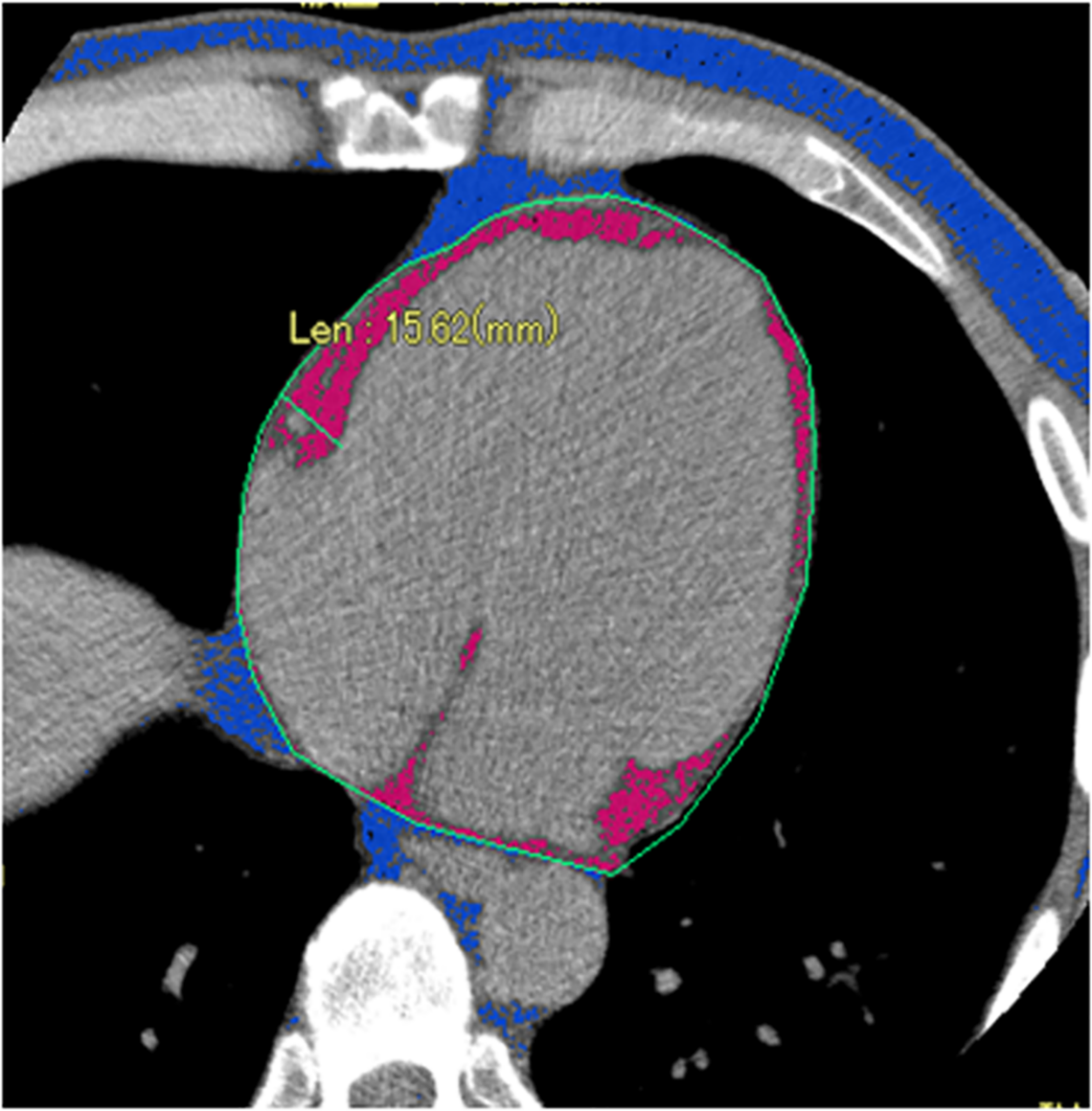
The representative image of CT image evaluating EAT. A region of interest (ROI) was manually traced along the visceral pericardium as indicated green line. A density range between -200 and -30 Hounsfield Units was used to isolate adipose tissue displayed as pink area.

Measurements of EAT were performed by CT scanning with cross-sectional axial views employing imageswith 3mm gaps. The range for measurement of EAT was set as the origin of the left main coronary trunk for the superior border and 6cm below the superior border for the basal border [15]. Quantification of the EAT area (cm^2^) was performed using software programs (Slim Vision 5, Cybernet Systems, Japan). The EAT area was calculated by manually tracing a region of interest (ROI), which was placed outside the line of the visceral pericardium to exclude pericardial fluid. A density range between -200 and -30 Hounsfield Units was used to isolate adipose tissue [14, 16]. The EAT area of each slice was summed from 20 slices and multiplied by the slice number to evaluate the EAT volume (cm^3^). A representative image is shown in Fig 1. The EAT values obtained from 20 slices were validated by assessing the correlation with those of whole cardiac scanning image from the same person, in a portion of the subjects enrolled in this study (n = 105, r = 0.974, p < 0.01).

### CT imaging analyses

The total CACS were analyzed according to the Agatston method [15] and were determined as previously reported [17]. The volume of abdominal fat, divided into visceral fat area and subcutaneous fat area, was obtained from CT images scanned at the level of the fourth lumbar vertebra [18]. Hepatic steatosis was defined as a liver to spleen density ratio below 0.9, based on plain abdominal CT [19].

### Measurements of ABI, baPWV and carotid artery IMT

The ABI (ankle brachial pressure index) and brachial ankle pulse wave velocity (baPWV) were measured using an automatic waveform analyzer (BP-203RPE; Colin Co., Komaki, Japan). The intima-media thickness (IMT) of the carotid arteries was measured using ultrasound diagnostic equipment (LOGIQ 500, GE Yokogawa Medical Systems Corp., Hino, Tokyo, Japan) and the max IMT, i.e. the thickest portion detected in the scanned regions, was determined as described previously [17]. These measurement values and CACS were evaluated in order to screen for asymptomatic atherosclerosis in T2DM patients.

### Laboratory data analysis

Laboratory values were measured employing routine techniques on blood and urine samples obtained after a 12-h overnight fast in T2DM patients. The value of low dencity lipoprotein cholesterol (LDL-C) was measured using a direct assay method (Sekisui Medical Co., Tokyo, Japan). The serum levels of adipocytekines, including leptin and adiponectin, as well as those of oxidative stress markers, such as urinary 8-isoprostane and 8-hydroxydeoxyguanosine, and serum malondialdehyde-LDL cholesterol, and various unsaturated fatty acid were measured by SRL, Inc. (Tokyo, Japan).

Estimated glomerular filtration rates (eGFR) were calculated as shown below [20]. Serum creatinine (cre) based eGFR was defined as eGFRcre (mL/min/1.73m^2^) = 194 x cre^-1.094^ x age^-0.287^ (male), 194 x cre^-1.094^ x age^-0.287^ x 0.739 (female). Serum cystatin C (cys) based eGFR was defined as eGFRcys (mL/min/1.73m^2^) = (104 x cys^-1.019^ x 0.996 ^age^) - 8 (male), (104 x cys^-1.019^ x 0.996 ^age^ x 0.929) - 8 (female).

### Statistical analysis

Quantitative data are presented as means ± standard deviation (SD) or as medians with inter quartile range when the data showed a non-normal distribution. Comparisons between the subjects were performed employing the student *t* test and the chi-square test or, when the data showed a non-normal distribution, the Mann–Whitney *U*-test. The level of significance was set at *P* < 0.05. Multiple linear regression analyses were performed to evaluate parameters independently showing significant correlations with EAT and cystatin C. Clinical parameters, showing significant simple correlations with EAT or cystatin C, were assigned as independent variables in multivariate linear regression analysis, unless there was extreme collinearity. All statistical analyses were carried out using SPSS version 21 (SPSS Japan Inc., Tokyo, Japan).

## Results

The clinical characteristics of the 208 enrolled subjects are shown in Table 1. Mean age was 58 years, mean diabetes duration was 9.7 years and 125 subjects were males. The mean body mass index (BMI), visceral fat area and homeostasis model assessment (HOMA) -R were 27.0, 160.2 cm^2^ and 3.1, respectively, indicating moderately obesity and the presence of insulin resistance as compared to Japanese subjects with T2DM in general. The mean EAT values was 52.4 cm^3^.

**Table 1:** Baseline characteristics of the study subjects.

	n = 208
Gender (male / female)	125 / 83
Age (years)	58.0 ± 14.3
BMI (kg/m^2^)	27.0 ± 6
Diabetes duration (years)	9.7 ± 9.6
Hypertension, n (%)	115 (55)
Dyslipidemia, n (%)	152 (73)
SBP (mmHg)	127.8 ± 19.2
DBP (mmHg)	76.0 ± 12.8
Total cholesterol (mg/dL)	193.9 ± 49.3
Triglyceride (mg/dL)	150.8 ± 92.5
HDL cholesterol (mg/dL)	46.1 ± 13.8
LDL cholesterol (mg/dL)	119.3 ± 38.7
eGFRcre (mL/min/ 1.73 m^2^)	74.8 ± 15.2
eGFRcys (mL/min / 1.73 m^2^)	81.1 ± 22.6
24hrs creatinine clearance (mL/min)	87.3 ± 30.1
Cystatin C (mg/L)	0.96 ± 0.24
Fasting blood glucose (mg/dL)	172.7 ± 79.1
HbA1c (%)	10.4 ± 2.4
HOMA-R	3.1 ± 2.9
C-peptide (ng/mL)	1.64 ± 1.00
Urinary 8-isoprostane (pg/mgCr)	245.8 ± 124.7
Urinary 8-OHdG (pg/mgCr)	11.1 ± 5.5
MDA-LDL (U/dL)	134.5 ± 47.5
Leptin (ng/mL)	12.2 ± 9.6
Adiponectin (μg/mL)	3.2 ± 3.3
High-sensitivity C-reactive protein (mg/dL)	0.11 (0.04–0.31)
DGLA (μg/mL)	37.6 ± 18.8
AA (μg/mL)	193.8 ± 58.7
EPA (μg/mL)	73.5 ± 46.7
DHA (μg/mL)	149.8 ± 59.6
EPA/AA	0.4 ± 0.3
max IMT (mm)	1.45 (1.00–2.00)
baPWV (cm/s)	1499 (1251–1749)
ABI	1.12 (1.06–1.18)
Coronary artery calcification score, (AU)	20.0 (0–143.8)
Visceral Fat Area (cm^2^)	156.4 (112.8–200.0)
Subcutaneous Fat Area (cm^2^)	175.0 (121.2–267.1)
EAT (cm^3^)	52.4 ± 29.5
Liver spleen ratio	1.12 ± 0.28
Diabetic retinopathy, n (%)	58 (28)
Peripheral neuropathy, n (%)	94 (46)
History of smoking, n (%)	110 (53)
Af, n (%)	18 (9)
History of CVD, n (%)	25 (12)
Family history of CVD, n (%)	82 (39)
Diabetic nephropathy Normoalbuminuria (<30 mg/ gCre)	140 (77.4)
Microalbuminuria (30–299 mg/ gCre)	51 (24.5)
Overtalbuminuria (≥300 mg/ gCre)	17 (8.1%)
DPP-4 inhibitors, n (%)	101 (49)
Insulin, n (%)	96 (46)
Metformin, n (%)	53 (25)
Sulfonylurea, n (%)	37 (18)
Alpha-glucosidase inhibitor, n (%)	30 (14)
Glinide, n (%)	6 (3)
Glucagon-like peptide-1, n (%)	9 (4)
Thiazolidinedione, n (%)	13 (6)
SGLT inhibitor, n (%)	5 (2)
Statins, n (%)	86 (41)
RAS inhibitors, n (%)	84 (40)
Calcium channel blocker, n (%)	57 (27)
Diuretics, n (%)	31 (15)

SBP: systolic blood pressure, DBP: diastolic blood pressure, HbA1c: hemoglobin A1c, DGLA: dihomo-gamma-linolenic acid, AA: arachidonic acid, EPA: eicosapentaenoic acid, DHA: docosahexaenoic acid, CVD: cerebral vascular disease, DPP: dipeptidyl peptidase, RAS: renin-angiotensin system, SGLT: Sodium-dependent glucose transporter, 8-OHdG: 8-hydroxydeoxyguanosine

The volume of EAT correlated positively with age (r = 0.206, p < 0.01), BMI (r = 0.488, p < 0.01), visceral fat area (r = 0.603, p < 0.01), levels of serum dihomo-gamma-linolenic acid levels (DGLA) (r = 0.184, p < 0.01), leptin (r = 0.496, p < 0.01), cystatin C (r = 0.320, p < 0.01) and C-peptide (r = 0.263, p < 0.01), as well as with HOMA-R (r = 0.262 p < 0.01) (Table 2). The EAT values showed negative correlations with adiponectin (r = -0.173, p < 0.05), eGFRcre (r = -0.218, p < 0.01), eGFRcys (r = -0.362, p < 0.01) and the liver-to-spleen ratio (r = -0.186, p < 0.01). Consistent with previous reports, EAT values correlated with the parameters known to be related to metabolic syndrome in Japanese subjects. In addition, the EAT values were higher in females and in the subjects with hypertension.

**Table 2:** Correlations of clinical parameters with EAT.

Variable	Correlation coefficient	EAT values	*P* value
Age, (years)	0.206		0.003
BMI (kg/m^2^)	0.488		< 0.001
Diabetes duration (years)	0.111		0.114
SBP (mmHg)	0.080		0.253
DBP (mmHg)	-0.006		0.929
Total cholesterol (mg/dL)	-0.077		0.266
Triglyceride (mg/dL)	0.045		0.516
HDL cholesterol (mg/dL)	-0.123		0.076
LDL cholesterol (mg/dL)	-0.048		0.487
eGFRcre (ml/min /1.73 m^2^)	-0.218		0.002
eGFRcys (ml/min / 1.73 m^2^)	-0.362		< 0.001
24hrs creatinine clearance (mL/min)	0.011		0.877
Cystatin C (mg/L)	0.320		< 0.001
Fasting blood glucose (mg/dL)	-0.079		0.258
HbA1c (%)	-0.112		0.110
HOMA-R	0.262		< 0.001
C-peptide (ng/mL)	0.264		< 0.001
Urinary 8-isoprostane (pg/mgCr)	-0.029		0.681
Urinary 8-OHdG (pg/mgCr)	0.024		0.728
MAD-LDL (U/dL)	0.018		0.795
Leptin (ng/mL)	0.496		< 0.001
Adiponectin (μg/mL)	-0.173		0.012
High-sensitivity C-reactive protein (mg/L)	0.137		0.062
DGLA (μg/mL)	0.184		0.008
AA (μg/mL)	-0.010		0.890
EPA (μg/mL)	0.095		0.171
DHA (μg/mL)	0.039		0.580
EPA/AA	0.090		0.194
max IMT (mm)	-0.013		0.856
baPWV (cm/s)	0.095		0.173
ABI	0.066		0.344
Coronary artery calcification score (AU)	0.074		0.288
Visceral Fat Area (cm^2^)	0.603		< 0.001
Liver-to-spleen ratio	-0.186		0.009
Male vs Female*		48.5±27.2 vs58.5±32.0	0.022
Hypertension (Yes vs No)*		55.8±29.0 vs 48.3±29.9	0.048
Dyslipidemia (Yes vs No)*		51.7±28.0 vs 54.5±33.5	0.577

Spearman rank correlation coefficient

* analyzed by Mann-Whitney U-test, values are mean±SD

Next, we performed multiple linear regression analyses to identify variables independently related to EAT values (Table 3). Multivariate analysis, adjusted for sex, adiponectin, the liver-to-spleen ratio, DGLA and HOMA-R, revealed age, BMI, serum leptin level, the presence of hypertension and the cystatin C level to be positively related to EAT values. Since the serum cystatin C level is an established marker of renal function, to assess whether the effect of cystatin C on EAT values reflects glomerular filtration, we performed multivariate analysis employing Model 2, switching one of the dependent variables from cystatin C to eGFRcre. Intriguingly, this multiple linear regression analysis revealed eGFRcre to not be independently associated with EAT values. This result suggested the association between cystatin C and EAT values to be independent of glomerular filtration rate evaluated by serum creatinine level.

**Table 3:** Multiple regression analysis for EAT.

	Model 1.		Model 2.	
Variables	β	*P* value	β	*P* value
Age	0.335	< 0.001	0.373	< 0.001
Leptin	0.260	0.011	0.346	0.001
BMI	0.393	< 0.001	0.363	< 0.001
Cystatin C	0.199	0.003		
eGFRcre			-0.079	0.238

Model 1: independent variables: Age, Leptin, Sex, Adiponectin, Liver-to-spleen ratio, HOMA-R, BMI, DGLA, presence of hypertension and Cystatin-C

Model 2: independent variables: Age, Leptin, Sex, Adiponectin, Liver-to-spleen ratio, HOMA-R, BMI, DGLA, presence of hypertension and eGFRcre

β; the standard coefficient

the multiple coefficient of determination (R2) = 0.408 (Model 1) and 0.413 (Model 2)

To investigate the effects of cystatin C on clinical parameters in our study subjects, we performed simple and multiple linear regression analyses for cystatin C. The serum cystatin C level showed significant correlation with age, diabetes duration, visceral fat area, EAT, IMT, PWV and CACS (Table 4). Furthermore, cystatin C correlated negatively with the parameters reflecting renal function, including eGFRcre, eGFRcys and 24hrCcr, and HbA1c. The EAT values were higher in the subjects with hypertension. Interestingly, cystatin C showed associations with the parameters related to metabolic syndrome and with the surrogate markers of atherosclerosis. Multiple linear regression analysis, adjusted for age, gender, EAT, max IMT, CACS, baPWV, HbA1c and the presence of hypertension, revealed age, male, EAT and the presence of hypertension to show independent statistically significant associations with cystatin C (Table 5).

**Table 4:** Correlations of clinical parameters with cystatin C.

Variable	Correlation cofficient	Cystatin C value	*P* value
Age (years)	0.450		< 0.001
BMI (kg/m^2^)	-0.040		0.565
Diabetes duration (years)	0.290		< 0.001
SBP (mmHg)	-0.059		0.394
DBP (mmHg)	0.390		0.775
Total cholesterol (mg/dL)	-0.045		0.516
Triglyceride (mg/dL)	0.122		0.080
HDL cholesterol (mg/dL)	-0.130		0.062
LDL cholesterol (mg/dL)	-0.102		0.141
eGFRcre (ml/min/ 1.73 m^2^)	-0.759		< 0.001
eGFRcys (ml/min/ 1.73 m^2^)	-0.963		< 0.001
24hrs creatinine clearance (mL/min)	-0.489		< 0.001
Fasting blood glucose (mg/dL)	-0.166		0.017
HbA1c (%)	-0.204		0.003
HOMA-R	-0.026		0.712
C-peptide (ng/mL)	0.183		0.008
Urinary 8-isoprostane (pg/mgCr)	-0.086		0.223
Urinary 8-OHdG (pg/mgCr)	0.063		0.367
MDA-LDL (U/dL)	-0.027		0.703
Leptin (ng/mL)	0.090		0.196
Adiponectin (μg/mL)	0.074		0.290
High-sensitivity C-reactive protein (mg/L)	0.138		0.060
DGLA (μg/mL)	0.084		0.228
AA (μg/mL)	-0.147		0.034
EPA (μg/mL)	0.151		0.029
DHA (μg/mL)	0.093		0.179
EPA/AA	0.214		0.002
max IMT (mm)	0.285		< 0.001
baPWV (cm/s)	0.340		< 0.001
ABI	0.065		0.351
Coronary artery calcification score (AU)	0.320		< 0.001
Visceral Fat Area (cm^2^)	0.199		0.006
Subcutaneous Fat Area (cm^3^)	-0.057		0.440
EAT (cm^3^)	0.320		< 0.001
Liver-to-spleen ratio	0.058		0.445
Male vs Female*		0.98±0.26 vs 0.92±0.20	0.138
Hypertension (Yes vs No)*		1.01±0.27 vs 0.89±0.18	<0.001
Dyslipidemia (Yes vs No)*		0.97±0.25 vs 0.93±0.22	0.361

Spearman rank correlation coefficient

* analyzed by Mann-Whitney U-test, values are mean±SD

**Table 5:** Multiple regression analysis for cystatin C.

Factors	β	*P* value
Age	0.243	0.005
Sex	-0.188	< 0.001
EAT	0.306	< 0.001
max IMT	0.074	0.334
CACS	0.042	0.559
PWV	-0.045	0.576
Hypertension	0.142	0.039
HbA1c	-0.043	0.525

β; the standard coefficient

the multiple coefficient of determination (R2) = 0.259

In addition, there were no significant differences either EAT volume or cystatin C values between users and non-users of various medications, including drugs for diabetes, statins and renin-angiotensin system inhibitors.

## Discussion

This study is the first, to our knowledge, to demonstrate a close relationship between EAT accumulation and the serum level of cystatin C, independent from glomerular filtration rate in Japanese T2DM. Because the incidence of CAD in the Japanese population has been rising in recent decades, identification of factors contributing to the development of atherosclerosis resulting from adiposity is important. Our present observations shed light on the mechanism of atherosclerosis development in T2DM patients with fat accumulation.

EAT arises from brown adipose tissue as well as visceral adipose tissue [21] and possesses biological characteristics to similar those of visceral fat [22]. While the volume of EAT accounts for only one percent of whole fat mass [1], adipocytes in the epicardium are able to synthesize, produce and secrete bioactive humoral factors which are transported into the myocardium via vasocrine and/or paracrine pathways [23]. These bioactive molecules, including inflammatory cytokines, secreted from EAT might interact directly with coronary arteries and the myocardium. In addition, expressions and secretions of inflammatory cytokines, such as resistin, monocyte chemotactic protein (MCP)-1 and TNF-α, are higher in EAT than in subcutaneous fat tissue (SAT) [24] and inflammatory cells markedly infiltrate EAT as compared to SAT in CAD subjects [1]. Moreover, expressions of mRNA involved in oxidative stress are higher in EAT than in SAT [25]. Taken together, our results and those of other investigators indicate that EAT has distinctive pathogenic and pathophysiological characteristics with exacerbate inflammation and oxidative stress, leading to the development of atherosclerosis.

The volume of EAT reportedly showed association with surrogate markers of atherosclerosis, including CACS [5], carotid IMT [3], carotid stiffness [4] and the degree of coronary artery stenosis [26]. However, in this study, there was no association between EAT volume and surrogate markers of atherosclerosis, including CACS, max IMT, baPWV and ABI. A meta-analysis [27] and a multicenter study [28] also found no associations between EAT and atherosclerotic markers, such as CACS. In contrast, CACS was found to have a strong association with CAD and future cardiovascular events in subjects with chronic kidney disease (CKD) [29] and in a large prospective study [30]. Kaikita et al reported EAT to correlate positively not with calcified, but rather with non-calcified coronary plaque in subjects at high risk for CAD [31], suggesting that EAT may reflect early stage atherosclerosis and serve as a predictive marker of CAD progression. Statins administered to 41% of enrolled subjects, were reported to possibly impact the association between EAT and CACS, though whether statin usage promotes vascular calcification remains controvercial [32]. In addition, taking the T2DM population as a whole, wherein atherosclerosis development is attributable to multiple factors, we can reasonably specurate that the association between EAT and atherosclerotic markers might be obscured.

Cystatin C, a 13-kD endogenous cysteine proteinase inhibitor, is ubiquitously expressed, mainly in the brain, testis, lung, spleen and adipose tissue [33]. Cystatin C is freely filtered by the glomeruli, and catabolized in the proximal tubules. As muscle mass, gender and protein intake exert no influence on serum cystatin C, it is a more reliable marker of renal function than eGFR which is based on creatinine [34, 35].

Epidemiological studies have shown serum cystatin C to be increased in humans with obesity [36]. Naour et al showed cystatin C mRNA expression to be significantly elevated in omental and subcutaneous adipose tissue and increased three-fold in obese as compared to lean subjects [37]. Consistent with our result, a relationship between EAT and serum cystatin C was demonstrated in subjects with acromegaly [38]. Taken together with the previous findings of an association between visceral fat and EAT, cystatin C might be expressed in EAT and is then probably secreted into the circulation. Our present study demonstrated a strong association between EAT and serum cystatin C independent of glomerular filtration rate, supporting this hypothesis.

On the other hand, elevated cystatin C is reportedly associated with the presence or likely development of cardiovascular disease in subjects without chronic kidney disease [39, 40]. Moreover, the serum cystatin C level showed strong correlations with the degree of CAD [41, 42] and all-cause mortality [43]. Taken together, these results indicate that cystatin C is not simply a marker of impaired kidney function but also a marker of cardiovascular disease. Cystatin C is an endogenous inhibitor of cysteine protease, including cathepsin B, K and S, which are involved in degradation of the extracellular matrix and migration of monocytes and macrophages into the intima [44]. An imbalance between cysteine proteases and their inhibitor, cystatin C, may affect vascular inflammation, potentially leading to the development of atherosclerosis and inflammatory disorders [45]. Associations of serum cystatin C with inflammatory parameters, C-reactive protein and fibrinogen, were demonstrated, suggesting a role of cystatin C in systemic inflammation [46]. Furthermore, plasma cystatin C levels correlated with a build-up of amyloid deposits in the vascular walls in myocardial ischemic model mice [47]. In this study, elevated serum cystatin C showed a simple correlation with surrogate markers of atherosclerosis, such as max IMT, baPWV and CACS, while these relationships disappeared with adjustment for age on multiple regression analysis. This result supports the hypothesis that cystatin C exerts an effect on atherosclerosis development in T2DM.

The strengths of this study include the employment of numerous surrogate markers for atherosclerosis and other obesity-related disease as independent variables in conducting correlation analyses focusing on EAT. Among the obesity-related markers, such as adipocytokines, inflammatory cytokines and polyunsaturated fatty acids, studied herein, a particularly strong association between cystatin C and EAT was revealed. Serum cystatin C showed a significant association with EAT even after adjustment for several confounding factors. This study supports the hypothesis, and its clinical implications, that cystatin C elevation, related to EAT accumulation, exerts an additional impact on atherosclerosis development. Surrogate markers for evaluating atherosclerosis, including EAT volume, are informative but somewhat inconvenient and expensive to obtain, due to the equipment necessary for performing the measurement. In contrast, measuring serum cystatin C is non-invasive and low-cost. Therefore, assessment of serum cystatin C may allow early detection of atherosclerosis.

The major limitation of this study is its cross-sectional design, raising the possibility that our results show only associations. Therefore, the possible casual relationships between EAT and cystatin C cannot be confirmed, and further prospective study is required. Second, the extent of cardiac CT scanning for EAT evaluation is restricted, to within a range of 6cm from the origin of the left main coronary trunk. Third, the sample size was rather small for performing multivariate analysis incorporating large number of variables. In addition, despite the various clinical characteristics of the enrolled subjects varying rather markedly, the subjects were analyzed as a single group.

EAT accumulation and a high cystatin C concentration have been independently regarded as risk factors influencing atherosclerosis. This study showed a strong association between EAT and cystatin C independent of several confounders, including renal function parameters and several humoral factors. EAT accumulation may play an important role in Cystatin C secretion, thereby possibly contributing to cardiometabolic risk in T2DM.
